# Cardiometabolic risk status modifies the associations between remnant cholesterol with incident diabetes: evidence from two East Asian cohorts

**DOI:** 10.3389/fnut.2026.1770876

**Published:** 2026-06-01

**Authors:** Yibo Li, Zhongyin Chen, Lijing Shang, Yuhao Wang, Xinyi Liu, Yuehang Yang, Linfeng He

**Affiliations:** 1 The First Affiliated Hospital, and College of Clinical Medicine of Henan University of Science and Technology, Luoyang, China; 2 Department of Clinical Laboratory, Institute of Translational Medicine, Renmin Hospital of Wuhan University, Wuhan, China; 3 Department of General Medicine, Huazhong University of Science and Technology Hospital, Wuhan, China; 4 Department of Endocrinology, Union Hospital, Tongji Medical College, Huazhong University of Science and Technology, Wuhan, China; 5 Department of Cardiovascular Surgery, Beijing Aortic Disease Center, Beijing Anzhen Hospital of Capital Medical University, Beijing, China; 6 Department of Cardiology, Union Hospital, Tongji Medical College, Huazhong University of Science and Technology, Wuhan, China

**Keywords:** cardiometabolic risk factors, cohort study, diabetes mellitus, mediation analysis, remnant cholesterol

## Abstract

**Background:**

High remnant cholesterol (RC) frequently accompanies other traditional cardiometabolic risk factors (CMRF) during the progression of metabolic-related diseases. This study aims to differentiate the effects of traditional CMRF and to analyze the role of RC in the development of diabetes mellitus (DM).

**Methods:**

We performed a study using data from a Chinese (*n* = 112,694) and Japanese (*n* = 12,489) cohorts, stratified by CMRF status. The association between RC and DM was assessed using Cox regression models. Further exploration of the relationship was conducted using restricted cubic splines (RCS), extreme gradient boosting (XGboost), receiver operating characteristic (ROC) analysis, and mediation analysis. Sensitivity to unmeasured confounding was evaluated using E-value analysis.

**Results:**

RC was significantly associated with DM only in individuals with CMRF in both cohorts, and the association was nonlinear. In the Chinese cohort, DM risk increased when RC exceeded 0.5 mmol/L, while a saturation effect was observed in the Japanese cohort above 1.1 mmol/L. RC showed better predictive performance compared to LDL-C and total cholesterol in predicting DM risk among individuals with CMRF (AUC 0.606 and 0.647 in Chinese and Japanese cohorts, respectively). CMRF mediated 76.3 and 66.3% of the RC-DM association in the Chinese and Japanese cohorts.

**Conclusion:**

RC is nonlinearly associated with the incidence of DM in individuals with CMRF, primarily promoting its onset through the presence of CMRF.

## Graphical abstract

**Figure:**
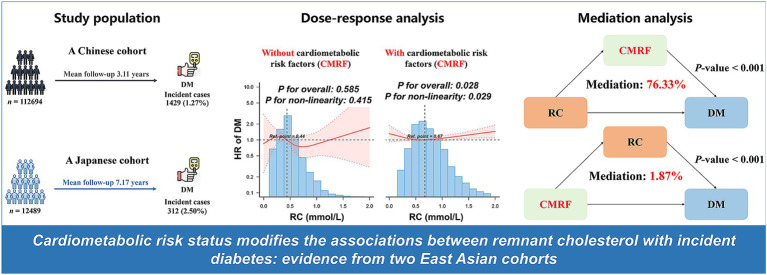

## 1 Introduction

Remnant cholesterol (RC), defined as the cholesterol content of triglyceride-rich lipoproteins, has recently gained increasing attention as a novel lipid biomarker closely associated with the risk of cardiovascular disease (CVD) and incident diabetes mellitus (DM) (1–4). RC reflects abnormalities in lipid metabolism beyond traditional cholesterol parameters and has been implicated in the pathogenesis of atherosclerosis and insulin resistance (5–9). Nevertheless, elevated RC frequently coexists with established cardiometabolic risk factors (CMRF) such as obesity, hypertension, and impaired glucose metabolism (10–14), making it challenging to disentangle whether the adverse effects attributed to RC are independent or mainly driven by these concurrent risk factors.

Currently, it remains unclear to what extent RC confers DM risk independently or through interactions with other CMRF. Most existing studies fail to stratify individuals by the presence or absence of CMRF (2–4), thereby limiting understanding of the differential impact of RC in diverse metabolic contexts. Clarifying this relationship is essential for more precise risk stratification and to guide targeted preventive interventions.

In this study, we leveraged data from two large cohort studies to investigate the association of RC with incident DM in individuals with and without CMRF. Our aims were to determine whether RC independently predicts DM risk across these distinct populations and to compare its predictive value against traditional lipid indices.

## 2 Materials and methods

### 2.1 Study population

**Figure 1:**
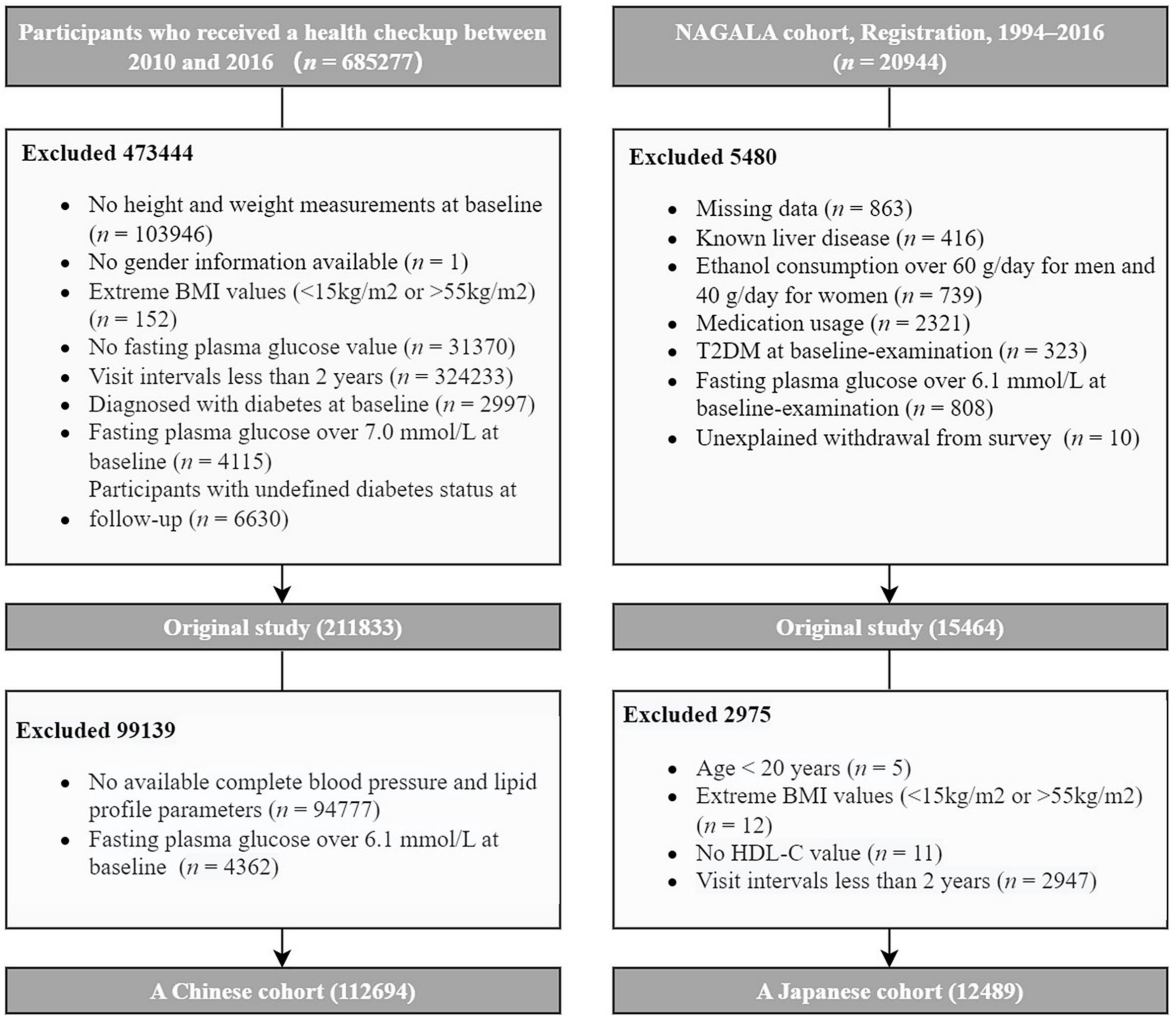
Flowchart for inclusion and exclusion of study participants.

This study utilized data from two large cohort studies: a Chinese cohort and a Japanese cohort. The Chinese cohort consisted of 112,694 participants (15), while the Japanese cohort included 12,489 participants (16). Both cohorts were divided into two groups based on the presence or absence of cardiometabolic risk factors (CMRF). The inclusion criteria were adults aged 20 years or older, with available baseline data on sociodemographic, anthropometric, and biochemical measures. Participants with a history of DM or those who had missing data on essential covariates were excluded from the analysis (Figure 1).

### 2.2 Data collection

Data were collected during baseline assessments and follow-up visits. Baseline data included information on age, sex, smoking status, alcohol consumption, anthropometric measurements (such as body mass index, BMI), and biochemical markers (including lipids, glucose, and liver enzymes). Smoking status and alcohol consumption were recorded as categorical variables: never, past, or current use. Additionally, fasting plasma glucose (FPG) and lipid profiles were measured after at least 8 h of fasting. Follow-up data were collected from medical records or direct follow-up visits at regular intervals.

### 2.3 Definition of CMRF

CMRF were defined based on the presence of one or more of the following conditions: obesity (BMI ≥ 23 kg/m^2^), hypertension (systolic blood pressure ≥130 mmHg or diastolic blood pressure ≥ 85 mmHg), (triglycerides ≥1.7 mmol/L), and impaired fasting glucose (FPG ≥ 5.6 mmol/L). Participants who met at least one of these criteria were classified as having CMRF (17).

### 2.4 Definition of RC

RC was calculated as the difference between total cholesterol (TC) and the sum of high-density lipoprotein cholesterol (HDL-C) and low-density lipoprotein cholesterol (LDL-C) (18).

### 2.5 Statistical analysis

Descriptive statistics were used to summarize baseline characteristics of the study population. Continuous variables were expressed as mean (standard deviation, SD), and categorical variables were expressed as frequency (percentage). Comparisons between groups (with and without CMRF) were made using independent *t*-tests for continuous variables and chi-squared tests for categorical variables.

The primary analysis involved Cox proportional hazards models to evaluate the association between RC and the risk of incident DM. Models were adjusted for age, sex, smoking status, and alcohol consumption. The restricted cubic spline (RCS) method was employed to explore the nonlinear relationship between RC and DM risk. Extreme gradient boosting (XGboost) and receiver operating characteristic (ROC) analysis were used to assess the predictive performance of RC for DM risk, with the area under the curve (AUC) calculated for each lipid parameter. Mediation analysis was conducted using the mediation package for R to examine the indirect and direct effects of CMRF in the relationship between RC and DM. Sensitivity analysis using E-values was performed to assess the potential impact of unmeasured confounding on the results.

Statistical significance was set at a *p*-value of <0.05. All analyses were performed using EmpowerStats (X&Y solutions, Inc., Boston, MA)^1^ and R version 4.2.0 (R Foundation for Statistical Computing, Vienna, Austria).

## 3 Results

### 3.1 Baseline characteristics of the study population

In both cohorts, individuals with CMRF were significantly older compared to those without, with mean ages of approximately 46 years versus 39 years in the Chinese cohort and around 45 years versus 42 years in the Japanese cohort (*p* < 0.001) (Table 1). Gender distribution differed notably between groups, with males more prevalent among individuals with CMRF in both cohorts (*p* < 0.001). Specifically, the proportion of males was about 65% in the Chinese cohort and 68% in the Japanese cohort among those with CMRF, whereas females predominated among those without CMRF. Participants with CMRF had significantly higher BMI, SBP, and DBP in comparison to those without CMRF (*p* < 0.001). The mean BMI was substantially higher in the CMRF groups (24.79 kg/m^2^ Chinese; 23.81 kg/m^2^ Japanese) versus the non-CMRF groups (around 20 kg/m^2^ in both cohorts). Similarly, SBP and DBP values were consistently elevated in those with CMRF.

**Table 1:** Baseline characteristics of study subjects by CMRF grouping.

Variable	Chinese cohort		*P-*value	Japanese cohort		*P-*value
	Without CMRF	With CMRF		Without CMRF	With CMRF	
*N*	39,414	73,280		5,698	6,791	
Age, year	38.78 (10.15)	46.25 (13.23)	<0.001	42.06 (8.30)	44.82 (8.76)	<0.001
Gender			<0.001			<0.001
Females	26,993 (68.49%)	25,790 (35.19%)		3,470 (60.90%)	2,188 (32.22%)	
Males	12,421 (31.51%)	47,490 (64.81%)		2,228 (39.10%)	4,603 (67.78%)	
BMI, kg/m^2^	20.42 (1.60)	24.79 (2.90)	<0.001	20.10 (1.68)	23.81 (3.01)	<0.001
SBP, mmHg	108.46 (10.36)	124.58 (16.35)	<0.001	107.09 (10.60)	120.57 (15.17)	<0.001
DBP, mmHg	67.89 (7.45)	77.59 (10.94)	<0.001	66.69 (7.69)	75.79 (10.48)	<0.001
FPG, mmol/L	4.70 (0.48)	4.98 (0.55)	<0.001	4.96 (0.33)	5.31 (0.40)	<0.001
TC, mmol/L	4.52 (0.81)	4.91 (0.91)	<0.001	4.98 (0.80)	5.23 (0.89)	<0.001
TG, mmol/L	0.84 (0.32)	1.62 (1.13)	<0.001	0.63 (0.30)	1.15 (0.76)	<0.001
HDL-C, mmol/L	1.47 (0.31)	1.32 (0.29)	<0.001	1.64 (0.35)	1.28 (0.34)	<0.001
LDL-C, mmol/L	2.58 (0.61)	2.85 (0.69)	<0.001	3.06 (0.72)	3.42 (0.80)	<0.001
RC, mmol/L	0.47 (0.32)	0.73 (0.45)	<0.001	0.29 (0.14)	0.52 (0.34)	<0.001
ALT, U/L	16.80 (18.30)	27.17 (22.42)	<0.001	16.01 (7.08)	23.57 (18.14)	<0.001
AST, U/L	21.39 (14.09)	25.23 (11.46)	<0.001	17.26 (5.89)	19.57 (10.62)	<0.001
Smoker			<0.001			<0.001
Never	8,546 (21.68%)	15,354 (20.95%)		3,896 (68.37%)	3,409 (50.20%)	
Past	238 (0.60%)	1,024 (1.40%)		791 (13.88%)	1,543 (22.72%)	
Current	1,184 (3.00%)	5,084 (6.94%)		1,011 (17.74%)	1,839 (27.08%)	
Missing data	29,446 (74.71%)	51,818 (70.71%)		–	–	
Drinker			<0.001			<0.001
No	8,759 (22.22%)	16,582 (22.63%)		4,630 (81.26%)	4,927 (72.55%)	
Yes	1,209 (3.07%)	4,880 (6.66%)		1,068 (18.74%)	1,864 (27.45%)	
Missing data	29,446 (74.71%)	51,818 (70.71%)		–	–	

Data are expressed as mean (SD) or n (%). CMRF cardiometabolic risk factors, BMI body mass index, SBP systolic blood pressure, DBP diastolic blood pressure, FPG fasting plasma glucose, TC total cholesterol, TG triglyceride, HDL-C high-density lipoprotein cholesterol, LDL-C low-density lipoprotein cholesterol, RC remnant cholesterol, ALT alanine aminotransferase, AST aspartate transaminase.

Lipid and glucose profiles also varied significantly. Participants with CMRF demonstrated higher FPG, TC, TG, LDL-C, and RC levels, and lower HDL-C levels, compared to their counterparts without CMRF (*p* < 0.001 for all). Of particular note, RC levels were markedly elevated in the CMRF groups, with average values of 0.73 mmol/L in the Chinese cohort and 0.52 mmol/L in the Japanese cohort, indicating potential clinical relevance. Additionally, individuals with CMRF were more likely to be current or past smokers and alcohol drinkers compared to those without CMRF.

### 3.2 Relationship between RC and incident DM among those with and without CMRF

**Figure 2:**
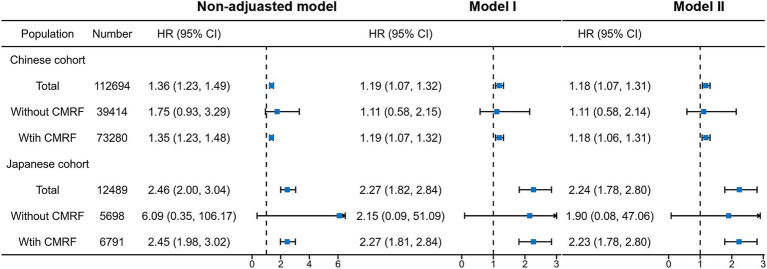
HRs of RC and incident DM. Model I: Adjusted for age and gender; Model II: Adjusted for age, gender, smoker, and drinker. HR hazard ratio, CI confidence interval, RC remnant cholesterol, CMRF cardiometabolic risk factors.

The association between RC and incident DM was consistently observed in both Chinese and Japanese cohorts. In the Chinese cohort, RC was significantly associated with increased risk of DM in the Non-adjusted model (HR = 1.36, 95% CI: 1.23, 1.49). After adjusting for age and gender (Model I), and further for smoking and drinking status (Model II), the association remained statistically significant (HR = 1.18, 95% CI: 1.07, 1.31). Stratified analysis revealed that this association was mainly driven by individuals with CMRF, for whom the adjusted HR remained significant (HR = 1.18, 95% CI: 1.06, 1.31). In contrast, among those without CMRF, the association was not statistically significant (HR = 1.11, 95% CI: 0.58, 2.14) (Figure 2). Of note, the extremely wide 95% confidence interval in the Japanese non-CMRF group (HR = 1.90, 95% CI: 0.08–47.06) suggests substantial statistical instability, likely due to the small number of incident DM events in this metabolically healthy subgroup; therefore, this estimate should be interpreted with caution. Although the HR in those without CMRF was elevated, the wide confidence interval indicated no statistical difference.

In the Japanese cohort, the relationship between RC and DM risk was even more pronounced. The Non-adjusted HR for the total population was 2.46 (95% CI: 2.00, 3.04), and remained significant after full adjustment in Model II (HR = 2.24, 95% CI: 1.78, 2.80). Similar to the Chinese cohort, the association was strongest among participants with CMRF (HR = 2.23, 95% CI: 1.78, 2.80) (Figure 2). Although the HR in those without CMRF was elevated (HR = 1.90), the wide confidence interval (95% CI: 0.08, 47.06) suggested no statistical difference.

**Figure 3:**
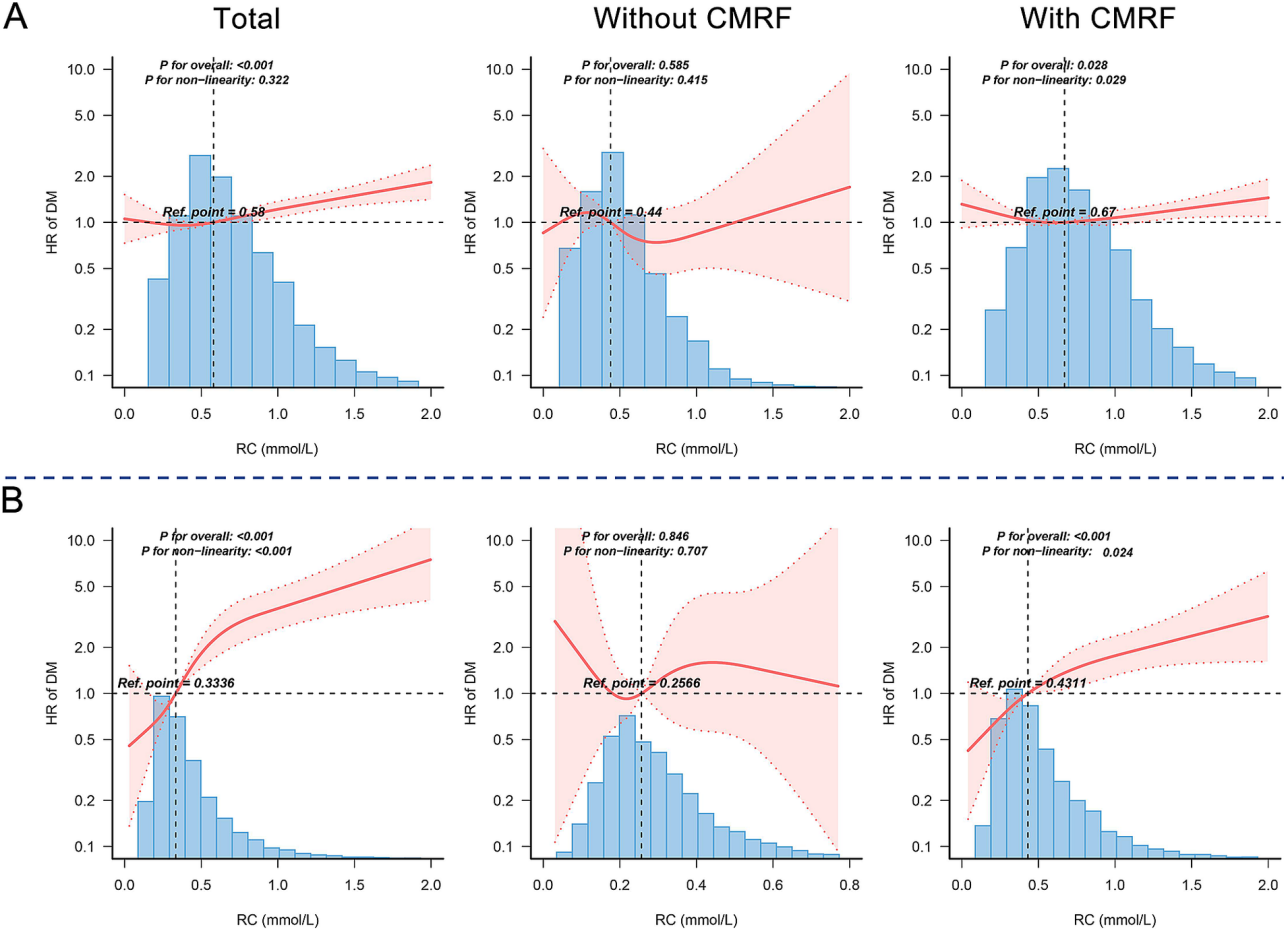
Restricted cubic spline (RCS) plot of RC and incident DM. **(A)** Chinese cohort. **(B)** Japanese cohort. All models were adjusted for age, sex, smoker, and drinker. RC, remnant cholesterol; DM, diabetes mellitus; CMRF, cardiometabolic risk factors.

RCS analyses further illustrated the dose–response relationship between RC and DM (Figure 3). In the Chinese cohort, RC showed a positive association with DM risk in the total population, although the overall trend was not statistically nonlinear (*P* for non-linearity = 0.322). In participants with CMRF, however, a significant nonlinear association was observed (*P* for non-linearity = 0.029), with a threshold RC level of approximately 0.5 mmol/L above which the DM risk increased markedly (Table 2). In the Japanese cohort, the nonlinear relationship was even more evident. Among those with CMRF, RC levels were positively and nonlinearly associated with DM risk (*P* for non-linearity = 0.024), with clear inflection points at 1.1 mmol/L. In contrast, the association was not significant among those without CMRF (*p* = 0.846).

**Table 2:** Inflection point segmented cox regression analysis of RC and DM in individuals with CMRF.

Variable	HR (95%CI)	
	Chinese	Japanese
Inflection point	0.5 mmol/L	1.1 mmol/L
< Inflection point	0.68 (0.42, 1.10) 0.115	3.76 (2.39, 5.90) < 0.001
≥ Inflection point	1.25 (1.12, 1.41) < 0.001	1.30 (0.75, 2.25) 0.345
*P* for log-likelihood test	0.035	0.008

All models were adjusted for age, gender, smoker, and drinker. RC, remnant cholesterol; DM, diabetes meliitus; CMRF, cardiometabolic risk factors; HR, hazard ratio; CI, confidence interval.

### 3.3 Efficacy of RC in predicting DM risk among those with CMRF

**Figure 4:**
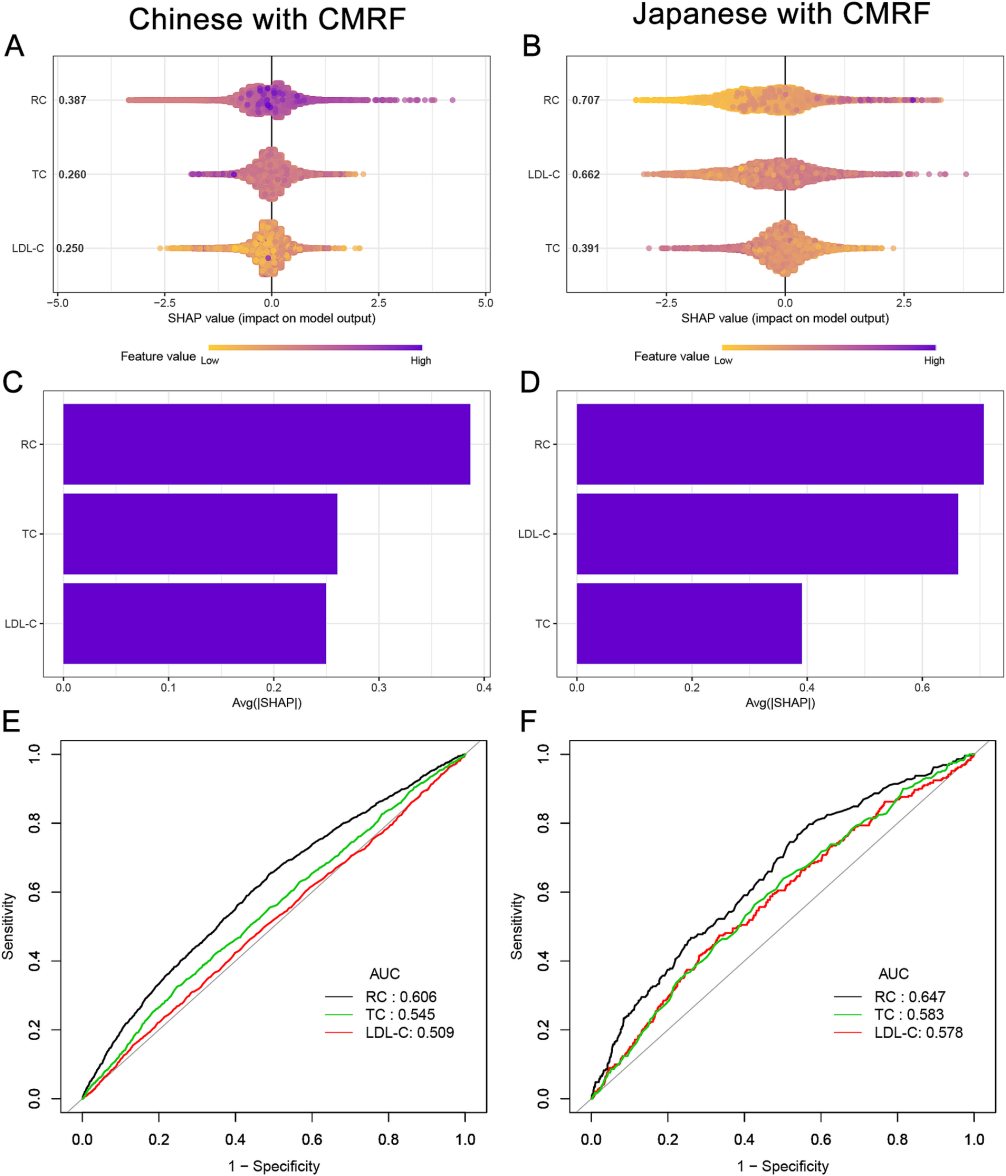
Predictive importance and diagnostic performance of RC and other lipids in individuals with CMRF. SHA*p* values of lipid features in XGBoost models for Chinese **(A)** and Japanese **(B)** cohorts. Average absolute SHAp values in Chinese **(C)** and Japanese **(D)** cohorts. ROC curves for RC, TC, and LDL-C in Chinese **(E)** and Japanese **(F)** cohorts. SHAP, Shapley additive explanations; ROC, receiver operating characteristic; AUC, area under the curve.

Among individuals with CMRF, RC showed relatively better predictive importance among the lipid markers tested, although its absolute discriminatory performance was modest (AUC 0.606 in the Chinese cohort and 0.647 in the Japanese cohort). Using SHAP values derived from an XGBoost-based DM prediction model, RC demonstrated the greatest impact on model output among the lipid parameters, with average absolute SHAP values of 0.387 and 0.707 in the Chinese and Japanese cohorts, respectively, exceeding those for TC and LDL-C (Figures 4A–D).

This finding was supported by ROC curve analysis, showing that RC achieved the greatest discriminatory performance with area under the ROC curve (AUC) values of 0.606 (95% CI, 0.591–0.621) in the Chinese cohort and 0.647 (95% CI, 0.615–0.678) in the Japanese cohort (Supplementary Table S2; Figures 4E,F). Optimal RC thresholds for DM prediction were 0.68 mmol/L in the Chinese group, yielding a sensitivity of 65.2% and specificity of 51.2%, and 0.39 mmol/L in the Japanese group, with higher sensitivity at 79.7% but lower specificity of 43.1%. We further compared the predictive performance of RC with BMI, a well-established metabolic risk factor. In individuals with CMRF, BMI alone yielded AUCs of 0.6353 (Chinese) and 0.6431 (Japanese); RC alone yielded AUCs of 0.606 and 0.647, respectively; and the combination of RC and BMI yielded AUCs of 0.6614 and 0.6849, indicating modest improvement over either marker alone (Supplementary Table S4). These results suggest that RC provides comparable predictive information to BMI, and combining both offers only marginal benefit. In contrast, LDL-C and TC showed lower AUCs, sensitivities, and specificities in both cohorts. These results indicate that RC is a more effective lipid biomarker than traditional cholesterol parameters for identifying individuals at elevated risk of developing DM among those with CMRF.

### 3.4 Mediation analyzes the relationship between RC, CMRF, and incident DM

**Figure 5:**
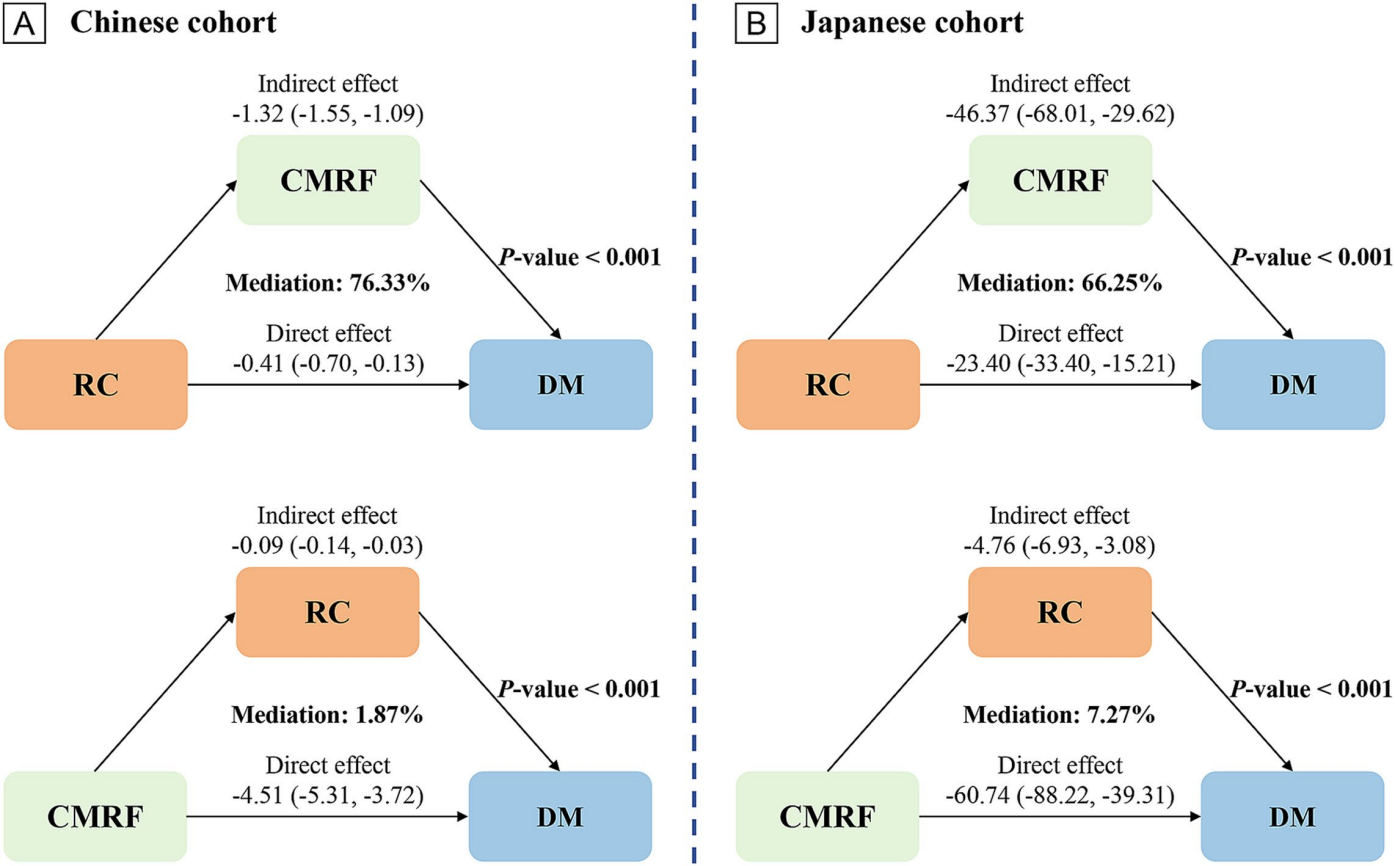
Mediation analysis illustrating the indirect and direct effects between RC, CMRF, and incident DM. **(A)** Chinese cohort; **(B)** Japanese cohort. All models were adjusted for age, sex, smoker, and drinker. DM, Diabetes mellitus; CMRF, cardiometabolic risk factors; RC, remnant cholesterol.

Mediation analysis revealed that the relationship between RC and incident DM was largely mediated by CMRF, particularly in the Chinese cohort. In this population, 76.33% of the total effect of RC on DM was mediated through CMRF, with a statistically significant indirect effect (−1.32, 95% CI: −1.55 to −1.09, *p* < 0.001). The direct effect of RC on DM, independent of CMRF, remained significant but accounted for only 23.67% of the total association (−0.41, 95% CI: −0.70 to −0.13, *p* = 0.006). The total effect of RC on DM in this cohort was −1.73 (95% CI: −2.09 to −1.36, *p* < 0.001), indicating a strong overall link with DM risk, predominantly mediated through CMRF (Figure 5A and Supplementary Table S3).

In the Japanese cohort, a similar but slightly attenuated mediation pattern was observed. CMRF accounted for 66.25% of the total effect of RC on DM (indirect effect = −46.37, 95% CI: −68.01 to −29.62, *p* < 0.001), while the direct effect contributed 33.75% (−23.40, 95% CI: −33.40 to −15.21, *p* < 0.001), leading to a total effect of −69.77 (95% CI: −99.38 to −47.61, *p* < 0.001) (Figure 5B and Supplementary Table S3). These results indicate that RC influences DM risk both directly and indirectly via its impact on CMRF.

Additionally, reverse mediation models—assessing whether RC mediated the effect of CMRF on DM—revealed much smaller mediation proportions. In the Chinese cohort, only 1.87% of the effect of CMRF on DM was mediated through RC (indirect effect = −0.09, 95% CI: −0.14 to −0.03, *p* = 0.004). Similarly, in the Japanese cohort, RC mediated 7.27% of the total effect (indirect effect = −4.76, 95% CI: −6.93 to −3.08, *p* < 0.001). These findings reinforce the notion that CMRF plays a central role in the causal pathway linking RC to incident DM, rather than the reverse.

### 3.5 Sensitivity analysis

To assess the robustness of the observed associations between RC and incident DM to potential unmeasured confounding, E-value analyses were conducted among individuals with CMRF in both the Chinese and Japanese cohorts (Supplementary Figure S1). In the Chinese cohort, the E-value for the observed association between RC and DM was 1.64, with a lower confidence limit E-value of 1.31. This suggests that an unmeasured confounder would need to be associated with both RC and DM by a risk ratio of at least 1.64, independent of the measured covariates, to fully explain away the observed effect.

In contrast, the Japanese cohort showed a substantially higher E-value of 3.89, with a lower confidence bound of 2.96. This indicates a more robust association between RC and DM in this population, as it would take a considerably stronger unmeasured confounder to nullify the observed relationship. These results provide additional support for the validity of the findings, particularly in the Japanese cohort, and reinforce the role of RC as an independent risk factor for DM, especially in individuals with underlying cardiometabolic risk.

## 4 Discussion

This large-scale, multi-ethnic study demonstrates that the association between RC and incident DM is modified by CMRF status. Specifically, elevated RC significantly predicted DM development among individuals with existing CMRF in both Chinese and Japanese cohorts, whereas no significant association was observed in metabolically healthy individuals. Our analysis revealed nonlinear dose–response relationships with distinct threshold and saturation effects—approximately 0.5 mmol/L in the Chinese and 1.1 mmol/L in the Japanese CMRF populations—indicating that RC-related DM risk becomes apparent only beyond certain concentration thresholds and tends to plateau at a certain level. Mediation analyses further demonstrated that CMRF substantially mediated the RC–DM relationship in both populations, underscoring the central role of metabolic dysfunction in modulating RC’s diabetogenic impact.

These findings build upon prior research linking RC to cardiometabolic diseases, while offering novel insights into its interaction with metabolic risk states. For example, Varbo et al. previously demonstrated a link between elevated RC and coronary heart disease but did not assess how CMRF may modify this relationship (19). Similarly, Jørgensen et al. identified RC as an independent risk factor for myocardial infarction, without addressing its role in DM (20). Hu et al. later reported that RC posed a greater DM risk than LDL-C, but their cross-sectional design precluded causal inference (7). More recently, Huh et al. identified RC as a DM risk factor using a national cohort, although debates regarding the pathologic role of RC persist (4). The PROMINENT trial, for instance, found that pemafibrate reduced triglycerides and RC but did not lower ASCVD incidence, raising questions about RC’s causal role (21). Furthermore, data from the UK Biobank suggest paradoxical associations between reduced RC and increased atrial fibrillation risk (22).

Complicating interpretation, RC is strongly correlated with metabolic abnormalities such as obesity, hypertriglyceridemia, low HDL-C, and hypertension (10, 11, 13, 23), which may confound its apparent effects. Our previous work also highlighted the modifying influence of CMRF on the fatty liver–DM relationship (24). By stratifying participants by CMRF status, the present study helps resolve these inconsistencies, showing that RC predominantly increases DM risk in those with pre-existing metabolic disturbances. Moreover, the use of restricted cubic spline modeling revealed nonlinear risk patterns, with clearly defined thresholds and saturation plateaus, enhancing clinical interpretability of RC levels in DM risk assessment. Mediation analyses quantified the extent to which CMRF account for the RC–DM link, highlighting key intermediary pathways and identifying modifiable intervention targets.

From a mechanistic perspective, RC—comprising cholesterol within triglyceride-rich lipoprotein remnants such as very low-density lipoprotein and chylomicron remnants—contributes to systemic inflammation, endothelial dysfunction, and insulin resistance, all central to DM pathogenesis (25–28). RC activates macrophages and promotes secretion of pro-inflammatory cytokines (e.g., TNF-*α*, IL-6), fueling chronic low-grade inflammation (29, 30). Cohort studies have shown stronger associations between RC with systemic inflammation and DM than with LDL-C, consistent with our findings (7, 31). Additionally, RC induces oxidative stress in vascular endothelial cells, reducing nitric oxide bioavailability and impairing endothelial function, thereby promoting metabolic dysfunction (32, 33). Animal and cellular models have further demonstrated RC-induced ectopic lipid accumulation in pancreatic *β*-cells and hepatocytes, leading to lipotoxicity, impaired insulin secretion, and hepatic insulin resistance (34, 35). For instance, Pedrini et al. reported that β-cell exposure to RC-enriched serum reduced insulin synthesis and elevated local inflammation (36). These mechanisms may explain why RC does not independently trigger DM in metabolically healthy individuals but acts synergistically with existing metabolic dysfunction to promote disease. The estimated RC thresholds differed markedly between the two cohorts (0.5 mmol/L for Chinese vs. 1.1 mmol/L for Japanese). Several factors may contribute to this discrepancy. First, sample size imbalance (Chinese cohort with CMRF: *n* = 73,280; Japanese cohort with CMRF: n = 6,791) likely affects the precision and stability of threshold estimation; the larger Chinese cohort provides more robust estimates. Second, baseline RC levels differed (0.73 vs. 0.52 mmol/L), and the Japanese cohort’s higher threshold may reflect a later saturation effect due to dietary patterns (e.g., higher fish oil intake) or genetic polymorphisms (e.g., apolipoprotein E, lipoprotein lipase). Third, subtle differences in laboratory measurements or case ascertainment between the two studies cannot be excluded. Fourth, residual confounding from unmeasured lifestyle factors may differ in magnitude between populations. Therefore, while biological differences may contribute, methodological factors likely play a non-negligible role. The identified thresholds should be considered cohort-specific and require validation in other populations before clinical application.

This study offers several strengths. The inclusion of two well-characterized, large Asian cohorts improves the generalizability of our findings in East Asian populations, which are often underrepresented in metabolic research. Advanced analytical techniques—including Cox regression, restricted cubic splines, machine learning models (XGBoost with SHAP values), ROC analysis, and causal mediation analysis—allowed a comprehensive evaluation of direct, indirect, and nonlinear effects. Stratification by CMRF status permitted identification of critical effect modifiers often overlooked in prior studies. Sensitivity analyses using E-values further supported the plausibility of causal inferences, especially in the Japanese cohort. These methodological strengths provide robust, multidimensional evidence for RC’s contribution to DM risk and support its potential utility in clinical risk stratification. It should be noted, however, that the absolute predictive performance of RC as a standalone screening tool is modest (AUC < 0.70), and its value may lie more in improving pathophysiological understanding and risk stratification in combination with other metabolic markers rather than as a single test for DM screening. This nuance is important when considering clinical application.

However, several limitations should be acknowledged. First, sociodemographic variables such as educational level, socioeconomic status, and place of residence were not available in the original cohorts. Such factors may influence both lipid profiles and DM risk, and their absence may lead to residual confounding. Second, detailed lifestyle factors, including physical activity, dietary quality, and caloric intake, were not assessed in either cohort. Given the well-established association between lifestyle behaviors and DM risk, the lack of adjustment for these factors could bias our estimates. Third, it is important to note that E-values assess the impact of a single unmeasured confounder. The combined effect of multiple weaker confounders (e.g., physical inactivity, unhealthy diet, low education) could potentially attenuate the observed association, especially in the Chinese cohort where the E-value was modest (1.64). Thus, although our findings are robust to a single unmeasured confounder, we cannot completely rule out residual confounding from a constellation of unmeasured lifestyle and sociodemographic factors. The higher E-value in the Japanese cohort (3.89) provides some reassurance, but caution is warranted when interpreting the Chinese cohort results. Notably, the consistent findings between the Japanese cohort (which excluded all medicated participants) and the Chinese cohort (without medication data) argue against substantial confounding by lipid-lowering drugs, further strengthening the validity of our results. Fourth, in the Chinese cohort, data on lipid-lowering medication use (e.g., statins) were not available. Such medications may influence both RC levels and DM risk, potentially introducing residual confounding. Future studies should collect medication information to strengthen causal inference. Fifth, the lack of oral glucose tolerance tests and islet autoantibody data precluded definitive subtyping of incident DM; although the vast majority of cases in this adult population are presumed to be type 2 DM, we cannot completely exclude other DM types. Sixth, central obesity measures (e.g., waist circumference) were not available; only BMI was used as a general obesity marker. Finally, the identified RC thresholds should be considered cohort-specific and require validation in other populations before clinical application.

Future research should aim to replicate these findings in ethnically diverse populations and incorporate repeated measures of RC and metabolic traits to better capture dynamic interactions. Randomized controlled trials targeting RC reduction in metabolically high-risk individuals are essential to confirm causality and assess therapeutic efficacy. Concurrently, mechanistic investigations at cellular and molecular levels are needed to delineate RC’s effects on β-cell function and insulin signaling. Genetic studies may also clarify interethnic variations in RC thresholds. Lastly, integrating RC into multifactorial DM risk prediction models—alongside established and emerging biomarkers—could enhance personalized prevention strategies. Comprehensive intervention trials targeting RC and its downstream metabolic effects are critical to reducing the global burden of DM.

## 5 Conclusion

In conclusion, this study identifies RC as a significant and independent predictor of incident DM in individuals with established cardiometabolic risk. The strong mediation by CMRF underscores the central role of metabolic milieu in modulating RC’s diabetogenic effects. These findings support measuring RC as an adjunctive risk marker in metabolically vulnerable populations and justify targeted RC-lowering strategies within integrated cardiometabolic prevention frameworks.

## Data Availability

Publicly available datasets were analyzed in this study. This data can be found at: All datasets used in this study are publicly available in the Dryad digital repository (http://datadryad.org). The data can be accessed permanently via the following DOIs: https://doi.org/10.5061/dryad.ft8750v and https://doi.org/10.5061/dryad.8q0p192. No special permissions or restrictions are required to access these datasets.

## Footnotes

1
www.empowerstats.net

## Glossary

AUC: Area under the curve
ALT: Alanine aminotransferase
AST: Aspartate transaminase
BMI: Body mass index
CI: Confidence interval
CMRF: Cardiometabolic risk factors
CVD: Cardiovascular disease
DBP: Diastolic blood pressure
DM: Diabetes mellitus
FPG: Fasting plasma glucose
HDL-C: High-density lipoprotein cholesterol
HR: Hazard ratio
IL-6: Interleukin-6
LDL-C: Low-density lipoprotein cholesterol
RC: Remnant cholesterol
RCS: Restricted cubic spline
ROC: Receiver operating characteristic
SBP: Systolic blood pressure
SHAP: Shapley additive explanations
TC: Total cholesterol
TG: Triglyceride
TNF-*α*: Tumor necrosis factor-alpha
XGBoost: Extreme gradient boosting
