# Supplementary material for: Cardiometabolic risk status modifies the associations between remnant cholesterol with incident diabetes: evidence from two East Asian cohorts
Source: Front Nutr. 2026 Jun 1;13:1770876. doi: 10.3389/fnut.2026.1770876 (PMC13265384; doi:10.3389/fnut.2026.1770876)
Supplement: Supplementary file 1 [file Table_1.docx]

**Cardiometabolic risk status modifies the associations between remnant cholesterol with incident diabetes: evidence from two East Asian cohorts**

(Supplementary materials)

Table S1 Analysis of covariate covariance

|  | VIF | | | | | |
| --- | --- | --- | --- | --- | --- | --- |
|  | Chinese cohort | | | Japanese cohort | | |
|  | Total | Without CMRF | With CMRF | Total | Without CMRF | With CMRF |
| RC | 1.1 | 1 | 1 | 1.2 | 1.2 | 1.1 |
| Age | 1 | 1 | 1 | 1 | 1.1 | 1 |
| Gender | 1.1 | 1 | 1 | 1.5 | 1.5 | 1.4 |
| Smoker | 4.2 | 4.2 | 1.9 | 1.4 | 1.4 | 1.3 |
| Drinker | 4.1 | 4.2 | 1.7 | 1.2 | 1.2 | 1.1 |

VIF variance inflation factor, RC remnant cholesterol,

CMRF cardiometabolic risk factors.

Table S2 Efficacy of ROC curve analysis of cholesterol parameters to predict DM risk in individuals with cardiometabolic risk factors

|  | AUC | Best threshold | Specificity | Sensitivity |
| --- | --- | --- | --- | --- |
| Chinese cohrot |  |  |  |  |
| RC | 0.606 (0.5907, 06214) | 0.68 | 0.5119 | 0.6522 |
| LDL-C | 0.5095 (0.4937, 0.5253) | 2.895 | 0.5621 | 0.4628 |
| TC | 0.5452 (0.5295, 0.5609) | 5.465 | 0.753 | 0.325 |
| Japanese cohrot |  |  |  |  |
| RC | 0.647 (0.615, 06789) | 0.39 | 0.4305 | 0.7973 |
| LDL-C | 0.5778 (0.5439, 0.6117) | 3.7262 | 0.6658 | 0.4742 |
| TC | 0.5832 (0.5502, 0.6162) | 5.1849 | 0.4982 | 0.6392 |

ROC receiver operator characteristic, CMRF cardiometabolic risk factors, AUC area under the ROC curve, RC remnant cholesterol, LDL-C low-density lipoprotein cholesterol, TC total cholesterol.

Table S3 Mediation analyzes the relationship between RC, CMRF and DM

|  | Chinese cohort | | | Japanese cohort | | |
| --- | --- | --- | --- | --- | --- | --- |
| Effect | effect estimate (95%CI) | *P* | Mediation  (%) | effect estimate  (95%CI) | *P* | Mediation  (%) |
| RC → CMRF → DM | | | | | | |
| Indirect | -1.32 (-1.55 , -1.09) | <.001 | 76.33 | -46.37 (-68.01 , -29.62) | <.001 | 66.25 |
| Direct | -0.41 (-0.70 , -0.13) | 0.006 | 23.67 | -23.40 (-33.40 , -15.21) | <.001 | 33.75 |
| Total | -1.73 (-2.09 , -1.36) | <.001 | 100 | -69.77 (-99.38 , -47.61) | <.001 | 100 |
| CMRF → RC → DM | | | | | | |
| Indirect | -0.09 (-0.14 , -0.03) | 0.004 | 1.87 | -4.76 (-6.93 , -3.08) | <.001 | 7.27 |
| Direct | -4.51 (-5.31 , -3.72) | <.001 | 98.13 | -60.74 (-88.22 , -39.31) | <.001 | 92.73 |
| Total | -4.59 (-5.41 , -3.81) | <.001 | 100 | -65.50 (-93.88 , -43.08) | <.001 | 100 |

All models were adjusted for age, sex, smoker and drinker.

RC remnant cholesterol, DM diabetes meliitus, CMRF cardiometabolic risk factors, CI confidence interval.

Table S4 Efficacy of ROC curve analysis of RC and BMI to predict DM risk in individuals with cardiometabolic risk factors

|  | AUC | Best threshold | Specificity | Sensitivity |
| --- | --- | --- | --- | --- |
| Chinese cohrot |  |  |  |  |
| RC | 0.606 (0.5907, 06214) | 0.68 | 0.5119 | 0.6522 |
| BMI | 0.6353 (0.6195, 0.651) | 25.795 | 0.6766 | 0.5512 |
| RC+BMI | 0.6614 (0.6466, 0.6762) | -4.0097 | 0.6209 | 0.6315 |
| Japanese cohrot |  |  |  |  |
| RC | 0.647 (0.615, 06789) | 0.39 | 0.4305 | 0.7973 |
| BMI | 0.6431 (0.6077, 0.6786) | 25.9794 | 0.82 | 0.4296 |
| RC+BMI | 0.6849 (0.6511, 0.7187) | -3.041 | 0.7218 | 0.6014 |

BMI body mass index, ROC receiver operator characteristic, CMRF cardiometabolic risk factors, AUC area under the ROC curve, RC remnant cholesterol, LDL-C low-density lipoprotein cholesterol, TC total cholesterol.


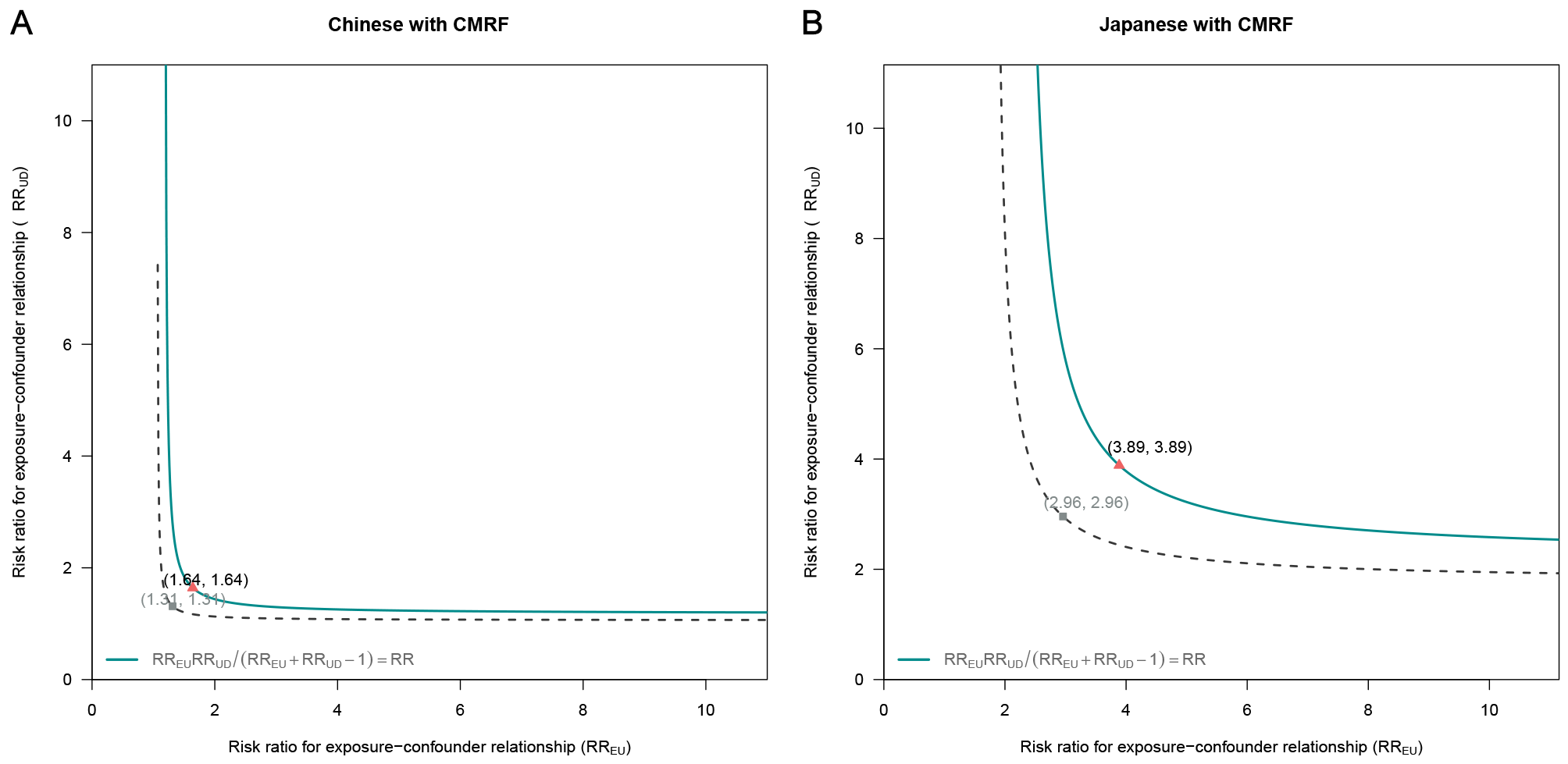


Figure S1 E-value assessment of RC and DM associations

A Chinese with CMRF; B Japanese with CMRF
